# Recurrent vaccine-strain varicella zoster virus reactivation in a child with acute lymphatic leukemia

**DOI:** 10.1016/j.idcr.2025.e02422

**Published:** 2025-11-05

**Authors:** Antonia Leutert, Aida Zeckanovic, Michael Huber, Patrick M. Meyer Sauteur, Raphael J. Morscher

**Affiliations:** a Division of Oncology, University Children's Hospital Zurich and Children's Research Center, University of Zurich, Zurich, Switzerland; b Institute of Medical Virology, University of Zurich, Zurich, Switzerland; c Division of Infectious Diseases and Hospital Epidemiology, Children’s Research Center, University Children’s Hospital Zurich, University of Zurich, Zurich, Switzerland; d Pediatric Cancer Metabolism Laboratory, Children's Research Center, University of Zurich, Zurich, Switzerland

**Keywords:** Varicella zoster, Oka vaccine strain, Immune compromised, Live-attenuated vaccine reactivation

## Abstract

This case illustrates recurrent herpes zoster (HZ) in a child with acute lymphatic leukemia. Interestingly, vaccine-strain HZ was confirmed by identifying the live-attenuated Oka vaccine strain (vOka) using metagenomic sequencing and sequence comparison at three loci that distinguish vOka from wild-type varicella zoster virus (VZV). Although vaccine-strain HZ is generally milder than HZ caused by wild-type VZV, prompt recognition and initiation of antiviral treatment is essential in immunocompromised patients, as fatal varicella due to disseminated vaccine-strain VZV has been reported in this high risk group.

## Case presentation

**Fig. 1:**
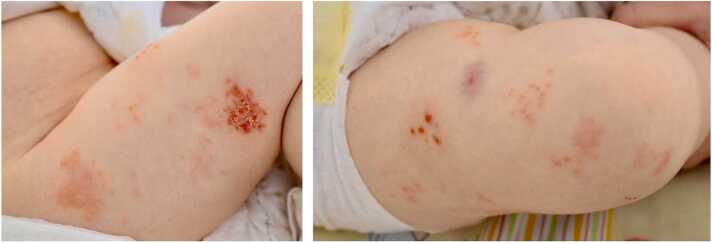
Rash consisting of grouped vesicles and crusts, partly on an erythematous ground, on the upper right leg (skin segments, L2–L5) on day 7 (left) and day 9 (right).

An 11-month-old child presented in good general condition with a rapidly progressing vesiculopapular rash on her right thigh. One month prior to this episode the patient had been diagnosed with infant B-cell precursor acute lymphoblastic leukemia (B-ALL). Intravenous acyclovir was initiated (1.5 g/m2/d) with the differential diagnosis of herpes simplex virus or varicella zoster virus (VZV) infection. The rash expanded over the next four days to form plaques involving the whole upper leg and the knee (Fig. 1).

VZV was detected by specific polymerase chain reaction (PCR) from vesical fluid and specific IgM and IgG antibodies in serum. A VZV-specific PCR in blood was negative. Upon continuous formation of new lesions, intravenous immunoglobulin (0.5 g/kg) was administered on day six. No new vesicles appeared after day seven. Intravenous acyclovir was administered for a total of ten days. Chemotherapy was reconvened on day 12.

Interestingly, the medical history revealed that the patient had been vaccinated with VZV at the site of the initial appearance of vesicles at the age of 9 months, according to the Swiss vaccination schedule. In Switzerland, vaccination against varicella (chickenpox) is routinely recommended for all infants, with two doses administered at 9 and 12 months of age [1]. The parents had also retrospectively reported that a generalized rash developed 10 days after vaccination.

Sequencing of the VZV strain revealed the presence of the live-attenuated Oka vaccine strain (vOka), confirming the diagnosis of vaccine-strain herpes zoster (HZ). Over the following two years, a total of seven HZ reactivations occurred, varying in severity.

## Discussion

VZV is an exclusively human herpes virus that causes chickenpox (varicella), becomes latent in cranial-nerve and dorsal-root ganglia, and frequently reactivates decades later to produce HZ [2]. HZ typically manifests as unilateral radicular pain and a vesicular rash that is generally limited to the dermatome innervated by a single dorsal root or cranial nerve ganglion [3].

HZ in children is relatively rare. Hope-Simpson estimated an incidence rate of 74 per 100,000 persons per year among children younger than 10 years of age. This rate increases substantially with age, reaching 1010 per 100,000 persons per year among those aged 80–89 years [4]. Overall, HZ affects approximately one in three immunocompetent people during their lifetime [3]. Immunocompromised children, however, have a 5–6 times higher risk of HZ [5]. In fact, recovery from varicella has been described to be associated with VZV-specific T cell-mediated immunity, which is also essential for limiting reactivation and replication of latent VZV, and thus for preventing HZ [3].

Viral genomics has identified five VZV clades and their geographical distribution: clades 1, 3, and 5 are of European origin; clade 2 includes Asian strains, such as vOka; and clade 4 contains African strains [6]. vOka was developed by Takahashi and colleagues in 1974 in Japan using an empirical attenuation approach involving the passage of P-Oka, a clinical isolate recovered from the skin lesions of a child with varicella, in cultured human and guinea pig cells [7]. Currently, three loci within open reading frame (ORF) 62 (nucleotide positions 106262, 107252, and 108111) are used to distinguish vOka from wild-type VZV [8]. In our patient, vOka was identified using metagenomic sequencing and sequence comparison at these three loci.

VZV is the only human herpes virus for which highly effective vaccines are available [6], and two doses of varicella vaccine are part of the national vaccination recommendations for children in many countries. Vaccine-strain HZ is extremely rare. The first documented case complicated by multiple recurrence was reported in 2008 [9]. vOka establishes latency in the dorsal root ganglion in the same manner as wild-type VZV, and reactivation can cause vaccine-strain HZ in both immunocompromised and immunocompetent individuals [6]. As was observed in our patient, vaccine-strain HZ rash in children tends to appear predominantly in the lumbar and sacral dermatome areas [10]. Moodley et al. predicted that, with respect to dermatomal localization of the viral eruption, HZ of the lumbar dermatomes in children is likely to be caused by the vaccine-strain, because HZ in those dermatomes is rare in children after wild-type VZV infection [11]. In contrast, HZ in children younger than 10 years caused by wild-type VZV most often involves thoracic dermatomes, which might be related to the intensity of the centrally distributed rash observed during varicella [10].

Bryant et al. showed that, although vOka can cause HZ in immunocompetent children, adolescents, and adults, this occurs at a much lower rate than HZ caused by wild-type VZV [12]. This has also been corroborated in immunocompromised patients: Hardy and colleagues showed that in children with leukemia who received the live-attenuated VZV vaccine subsequently experienced a lower incidence of HZ than those with wild-type VZV infection [13]. Importantly, it has been shown that immunization against varicella does not increase the incidence of HZ in high-risk groups [13]. Symptoms caused by vaccine-strain HZ were generally mild and resolved completely, suggesting that – although it is capable of causing central nervous system disease – this is a rare, self-limiting occurrence with typically milder symptoms in immunocompetent individuals [12]. However, single cases of fatal varicella due to disseminated vaccine-strain VZV have been reported in immunocompetent and immunocompromised children [14], [15].

Intravenous acyclovir treatment is recommended for immunocompromised patients with HZ, including patients being treated with high-dose corticosteroid treatment for more than 14 days [16]. Vaccine-strain VZV has been shown to have similar susceptibility to antivirals as wild-type VZV [17]. Although VZV immunoglobulin administered shortly after exposure can prevent or modify the course of the disease, it is not effective once the disease has developed [16].

## Conclusion

To our knowledge, this is the first case of recurrent HZ caused by vaccine-strain VZV in an infant with B-ALL. VZV vaccine is generally well tolerated. However, immunocompromised patients are at risk for developing dissemination or HZ following VZV vaccination. Although vaccine-strain HZ is generally milder compared to HZ caused by wild-type VZV, it is important to early recognize this adverse event and to initiate prompt antiviral treatment in order to prevent dissemination and sequelae in these high-risk situations.

## CRediT authorship contribution statement

**Leutert Antonia Laura:** Writing – original draft, Visualization, Conceptualization. **Aida Zeckanovic:** Writing – review & editing, Investigation, Data curation. **Michael Huber:** Writing – review & editing, Methodology, Investigation, Formal analysis. **Patrick M. Meyer Sauteur:** Writing – review & editing, Supervision, Formal analysis, Conceptualization. **Morscher Raphael Johannes:** Writing – review & editing, Visualization, Supervision, Funding acquisition, Conceptualization.

## Ethical approval

N/A.

## Consent

Written informed consent was obtained from the patient for publication of this case report and accompanying images. A copy of the written consent is available for review by the Editor-in-Chief of this journal on request.

## Funding

The authors were not paid for publishing or writing this article.

The authors have not been denied access to the study data and accept responsibility for its publication.

## Conflict of Interest

No conflicts of interest to declare.

## Declaration of Competing Interest

There are no competing interests to declare.
